# Differential Diagnosis of Autism Spectrum Disorder and Post Traumatic Stress Disorder: Two Clinical Cases

**DOI:** 10.3390/jcm7040071

**Published:** 2018-04-08

**Authors:** Katherine Kuhl-Meltzoff Stavropoulos, Yasamine Bolourian, Jan Blacher

**Affiliations:** University of California, Riverside, CA 92521, USA; ybolourian@gmail.com (Y.B.); jan.blacher@ucr.edu (J.B.)

**Keywords:** autism spectrum disorder, post-traumatic stress disorder, differential diagnosis

## Abstract

Autism spectrum disorder (ASD) is estimated to affect one in 68 children. Given the increase in both prevalence and awareness of ASD, it is critical to provide accurate and timely diagnosis. However, ASD often co-occurs with other disorders, making diagnosis difficult. The objective of the current case study was to provide two examples of differential diagnosis in ASD and post-traumatic stress disorder (PTSD) observed in an autism clinic. In both cases, the goal was to decide whether each child should be given a diagnosis of ASD, PTSD, or both.

## 1. Introduction

Autism spectrum disorder (ASD) is a developmental disorder characterized by impairments in social communication and the presence of restricted and repetitive behaviors and interests [1]. Current estimates of prevalence suggest that one in 68 children is diagnosed with ASD in the United States [2]. With growing awareness and prevalence of ASD, it is critical to correctly diagnose ASD in order to provide effective and timely intervention. However, accurate diagnosis can be difficult in cases where comorbid disorders are present. ASD often co-occurs with other disorders [3]. Children with ASD are likely to have psychiatric comorbidities, often including attention deficit/hyperactivity disorder (ADHD), anxiety disorders, and depression [4,5,6].

Anxiety disorders are among the most common comorbid disorders in ASD. Reports estimate that 40–45% of youth with ASD have a comorbid anxiety disorder [6,7]. Even without a formal diagnosis, anxiety-related behaviors are among the most common presenting problems for children with ASD in clinical settings [8]. A review of the literature suggested that between 11–84% of children with ASD experience anxiety that is considered impairing [6]. Post-traumatic stress disorder (PTSD) is characterized by avoidance, negative alterations in cognition and mood, reactivity, and intrusion symptoms, which occur in response to traumatic experiences. PTSD is no longer grouped with anxiety disorders in the 5th edition of the Diagnostic and Statistical Manual of Mental Disorders (DSM-5 [1], as it was in the 4th edition [9]. However, it remains with anxiety disorders in the International Statistical Classification of Diseases and Related Health Problems (ICD-10) [10]. Previous literature is mixed regarding the prevalence of comorbid ASD and PTSD. One previous study reported that 67% of children with ASD in an outpatient clinic in Istanbul demonstrated symptoms of PTSD, which was more than twice the rate observed in the general population [11]. However, other studies have reported rates of PTSD of 0–3% in youth with ASD seeking treatment [12,13]. Of note, in a large meta-analysis of studies on comorbid anxiety disorders in ASD [7], only two out of 86 provided data related to PTSD. This highlights the paucity of literature on the differential diagnosis and co-occurrence of PTSD and ASD.

Of critical importance to differential diagnosis, PTSD symptoms in children often involve both avoidant behavior and repetitive play themes [14,15]. As ASD is characterized by deficits in social communication (including lack of social initiation) and the presence of restricted and repetitive behavior, it can be difficult to parse whether these symptoms are due to PTSD versus ASD, or if a child should be given a dual diagnosis. Previous literature related to PTSD and ASD is limited. Manuscripts in recent years call for increases in research into the overlap between PTSD and ASD [16], highlight shared symptoms of PTSD and ASD [17], and hypothesize that ASD may be a risk factor for developing PTSD [18]. We found one case study discussing the difficulty of diagnosing PTSD in a child with a previous diagnosis of ASD [19]. Relevant to the current case study, the authors highlighted the complex interface between symptoms of ASD and PTSD, and ended up adding the diagnosis of PTSD to the pre-existing ASD diagnosis [19].

The goal of the current case study is to provide two illustrative examples of differential diagnosis in ASD and PTSD observed in an autism clinic. Parents of both children provided informed consent, and all procedures were approved by the Institutional Review Board of the University. In both cases, the children were referred to the clinic due to uncertainty about the cause of their symptoms, and discrepant information from school providers, psychologists, and other parents. In both cases, the goal was to decide whether each child should be given a diagnosis of ASD, PTSD, or both.

## 2. Case 1

### 2.1. Case Introduction

The patient was a 7-year-old boy, previously diagnosed with separation anxiety disorder. The family was from Southeast Asia and family members did not routinely speak English at home. Difficulties communicating in English negatively impacted the ability to get an accurate diagnosis. He presented to our clinic due to concerns about potential autism spectrum disorder or post-traumatic stress disorder.

### 2.2. Presenting Complaints

Current concerns included biting and hitting women, lack of interest in other children, inability to sit still, difficulty sleeping, and intense fears. At school, he was isolated and did not have friends. He did not follow directions, avoided eye contact, and was afraid of anything new. He had one younger brother.

### 2.3. History

The patient lived with his mother and younger brother. It was reported that as a very young child he was loving, affectionate and had strong emerging social-communication skills. However, around age two, his mother said he changed and seemed afraid. There was a history of physical and emotional abuse of both boys from their biological father starting at a young age. Their mother was unaware of the abuse for some time, but found out when the children brought it to her attention. The mother estimated that the abuse occurred for at least one year prior to her being aware of it. The two boys and their mother no longer lived with their father, and the parents were recently divorced. During the divorce, the court mandated psychological services for both children. Prior to the court order, the patient did not receive any psychological services. As part of the court ordered services, questions of diagnostic accuracy arose, particularly in relation to ASD and PTSD.

### 2.4. Assessment

Our assessment battery included gold-standard measures of autism (Autism Diagnostic Observation Schedule, Second Edition (ADOS-2); [20]), a cognitive assessment (Wechsler Preschool and Primary Scale of Intelligence, Third Edition (WPPSI-III); [21]), a measure of adaptive skills (Vineland Adaptive Behavior Scales, Second Edition (VABS-2); [22]), and a brief interview with the mother about family history. The parent interview also consisted of questions regarding information about abuse and trauma and her child’s behavior over time (e.g., what her son’s social skills looked like at a young age, when did the mother initially notice a change in behavior, and what occurred around that period of time).

### 2.5. Case Conceptualization

Although initially quiet, the patient warmed up to the examiner quickly when she asked about the toys he brought with him. Repetitive play themes related to families being in danger and a mother protecting her children were noted throughout the assessment. Whenever opportunities to engage in play arose, he made scenarios in which a mother was protecting her children from danger. Attempts to redirect to alternative play scenarios were largely unsuccessful, with the exception of a few violent play themes, in which he had characters hurt each other repetitively. Marked difficulties with communication were noted during the autism assessment, particularly related to language barriers and vocabulary. However, the examiner noted that he persisted in attempts to engage and augmented his verbal speech with eye contact and gestures in order to make himself understood. The cognitive test was unable to be completed due to difficulties with English. Although he was eager to engage in the presented tasks, he was unable to understand the presented directions in English, and had marked difficulty with all verbal items. Parent report indicated his adaptive skills were in the moderately low to low range. In particular, his socialization skills were in the low range as he did not engage with or seek out other children.

Despite difficulties in communication and the presence of repetitive behavior, our clinical determination was that a diagnosis of ASD would be inappropriate. Our determination was made in light of his strong efforts to engage in social communication despite his difficulty with English. For example, while trying to bring the examiner’s attention to a bird outside, he pointed, made eye contact, and used speech. When he was not able to explain something in English, he supplemented his speech with multiple types of gestures (e.g., hand gestures for “big” and “long”) and eye contact. His ADOS-2 comparison score was three, which fell into the “non-spectrum” classification. An important consideration for our clinical impression was his exposure to trauma, and the nature of his experiences. In light of his history with both physical and psychological abuse, and his mother filing for divorce and having custody of her sons, it was unsurprising that he evidenced repetitive play themes of danger and violence, and of a mother protecting her children. If this history were not known and documented, his repetitive play may have been confused for the repetitive behaviors often observed in ASD.

Regarding repetitive play themes, however, it is critical to note that children with ASD often have trouble with imaginative play, and with utilizing items in creative ways (e.g., pretending a block is a flower). Though our patient’s play had repetitive themes, it was functional and had imaginative aspects. For example, he utilized a box in our clinic as a place for his toy family to “hide” from danger. If children suffer trauma of some sort and present clinically with repetitive play themes related to violence or danger, but do not evidence repetitive behaviors or repetitive non-functional play (e.g., spinning wheels of a car), clinicians may wish to consider PTSD rather than ASD.

### 2.6. Complicating Factors

This case was complicated by a significant language barrier, and the patient’s young age. Due to his young age and difficulties communicating in English, we did not ask him questions about his past experiences related to trauma. Furthermore, it was unclear at times whether his difficulties with communication were due to a language barrier alone, or if anxiety was a contributing factor. Nonetheless, the patient clearly communicated that he understood much of what was asked through gestures and play.

### 2.7. Access and Barriers to Care

The family had multiple barriers to obtaining care. Most notable was that the mother was unsure of how and where to access services for her son due to the language barrier and lack of familiarity with US laws and regulations. She reported that both prior to and during the divorce the child’s biological father was adamantly against therapy, and she was unsure of the consequences of unilaterally seeking psychological services for her son. She was under significant financial stress, and was not sure how to find providers who either accepted her state insurance or offered free services. Finally, although the court ordered both children to receive therapy (which they were able to successfully obtain), she had trouble finding providers who could conduct a differential diagnosis of PTSD versus ASD.

### 2.8. Follow-Up

Approximately 11 months after being evaluated at our clinic, the patient’s mother was contacted to follow up on her son’s functioning. The mother seemed to articulate her situation much better at this time. After being seen at our clinic, the mother continued having difficulties finding appropriate services for her affected son. In particular, the language barrier continued to present a significant challenge, especially when meeting doctors and practitioners for the first time. As the mother sought therapy for her son for PTSD and related symptoms, multiple practitioners recommended he be re-evaluated for ASD, as his mother had difficulty explaining that he had already been evaluated and found to not have ASD. However, she reported that the most recent practitioner had been helpful, and understood the symptom overlap between PTSD and ASD, and was beginning treatment for PTSD-related symptoms. Additionally, she reported that her son’s difficulties with aggressive behaviors and peer relationships had improved. Overall, she expressed gratitude that she was able to obtain our clinical report, and she felt relief after the evaluation, as it was the first time a professional had explained the diagnostic overlap between PTSD and ASD, and she felt as though she was able to understand her son’s symptoms and why they might be occurring. She hoped that their current therapist would continue to be helpful and she expressed hope for the future.

## 3. Case 2

### 3.1. Case Introduction

The patient was a 6-year-old girl, previously diagnosed with ADHD and PTSD. She presented to our clinic for diagnostic clarification due to concerns about potential autism spectrum disorder.

### 3.2. Presenting Complaints

Current concerns included repetitive behaviors, such as spinning in circles, hand flapping, repeating words, and continuously quoting lines from movies. She was sensitive to noise and had intense tantrums, during which she would cry and throw herself to the floor in response to transitions or changes in routine or schedule. These tantrums occurred at least once per day. Her mother reported that she screamed in her sleep and had nightmares. She was reported to have no awareness of danger and to become overly attached to people she did not know well. Her mother described her behavior with others as being “extreme”, as she was either silent and refused to engage, or was inappropriately familiar. Socially, she did not play with other children for long, and experienced bullying at school.

### 3.3. History

The patient lived at home with her biological mother. Her biological mother had sole legal custody. She was an only child. There was a history of violent conflict between her biological mother and father, which she witnessed as a young child (prior to age 3). Her mother had difficulty remembering exactly how many instances of violence her daughter witnessed, and stated that she had difficulty remembering much about that period of time. There was a family history of autism on her mother’s side. The patient previously received speech and language services at school, but these were discontinued prior to our evaluation. At the time of our evaluation, the patient was receiving occupational therapy, and was taking Strattera for ADHD.

### 3.4. Assessment

Our assessment battery included gold-standard measures of autism [20], a cognitive assessment [21], a measure of adaptive skills [22], a measure of behavioral and emotional problems (Child Behavior Checklist (CBCL); [23]), and a brief interview with the mother about family and child behavioral history.

### 3.5. Case Conceptualization

The patient presented as extremely shy and anxious about visiting our clinic. She hid behind her mother’s legs and bit herself due to anxiety. However, the examiner was easily able to establish a rapport when talking about favorite colors and the color of her earrings. There was a marked difference in her demeanor as soon as she warmed up to the examiner, and she quickly became attached to all members of the clinical team. Similar to what her mother reported, we noticed she became particularly attached to multiple people, and appeared worried that everyone was “okay” and “taken care of”. For example, she repeatedly asked each person if they needed a snack, and she refused to eat her own snack until she had asked each person if they wanted any.

Her cognitive ability was in the average range, and she did not display any maladaptive or disruptive behaviors during the assessment. Despite her scores being solidly in the average range, she expressed worry that she did “badly” on the cognitive assessment, and told her mother that she was afraid the examiners thought she was “stupid.” Parent report of adaptive behaviors suggested her skills were in the moderately low to low range. Parent-reported internalizing (e.g., sad, anxious, and self-conscious) and externalizing (e.g., rule breaking, argumentative, and destructive) behaviors were clinically significant.

Socially, she demonstrated strong social communication skills. She was eager to engage with the examiners and used gestures, speech, and eye contact in order to express her feelings, thoughts, and ideas. She was able to have flexible back-and-forth conversations with the examiners about a variety of topics, and her language skills were age appropriate. She engaged in pretend play with the examiner and demonstrated creativity in her use of miniature play items.

Our overall clinical impressions were that the diagnosis of PTSD was appropriate, but an additional diagnosis of ASD was not warranted. Despite some socially-inappropriate behavior (e.g., becoming overly attached to members of our clinical team having met them only a short time prior), her social communicative skills and motivation to engage with others were strong. Additionally, she did not evidence areas of restricted interest or repetitive behaviors during the assessment. Her ADOS-2 comparison score was a two, which fell into the “non-spectrum” classification.

We note the importance of understanding her trauma history, as this helped put some of her anxious and attachment behaviors into the appropriate context. For example, her insistence that all adults were taken care of and “okay” prior to attending to her own needs and wants was not entirely surprising considering her history of witnessing interpersonal violence. Further, her attachment difficulties and tendency to either withdraw or become extremely clingy with adults were not unexpected for a young child who no longer has contact with a primary attachment figure (biological father). Interestingly, there has been a recent push to clearly differentiate between the social difficulties experienced in ASD and those observed in children with attachment disorders (e.g., reactive attachment disorder (RAD); disinhibited social engagement disorder (DSED)), as symptom overlap has been noted [24,25]. Though the DSM-5 and ICD-10 state that children cannot be diagnosed with both ASD and either RAD or DSED, Dickerson Mayes et al. [25] argue that the syndromes can co-exist and that ASD-specific symptoms (e.g., restricted interests, repetitive behaviors, and sensory hyper/hyposensitivity) reliably differentiate ASD from both RAD and DSED.

Also of interest are ongoing efforts to differentiate PTSD from both RAD and DSED [26,27], as all three are under the broader umbrella of “trauma-related disorders” in the DSM-5. One important factor in differentiating PTSD from RAD and DSED is case history. Whereas PTSD involves specific symptoms of intrusion, avoidance, negative alterations in cognition or mood, and alterations of arousal or reactivity following a traumatic event, RAD and DSED are often diagnosed in young children following extremes of insufficient care [27]. In this case, we did not consider either RAD or DSED due to the child not having a history of insufficient care and due to the presence of multiple PTSD symptoms.

### 3.6. Complicating Factors

The patient had multiple pre-existing diagnoses (PTSD and ADHD), which further complicated her ability to find and receive an accurate diagnosis. Although she had received a previous diagnosis of ADHD, our clinic does not test for or diagnose ADHD, and the presenting question did not pertain to ADHD. Often, children with existing mental health diagnoses do not receive an appropriate further diagnosis due to diagnostic overshadowing. Diagnostic overshadowing occurs when professionals attribute a patient’s symptoms to a particular condition while overlooking a co-occurring condition [28]. When diagnostic overshadowing occurs, it is difficult for parents or school professionals who are concerned about a child’s behavior to find providers who will conduct comprehensive diagnostic assessments. Although in this case the patient’s symptoms and presentation did not warrant a further diagnosis of ASD, it is important for providers to be aware of diagnostic overshadowing in order to avoid missing an accurate comorbid diagnosis.

A second complicating factor is the age at which the trauma occurred. When children experience or witness traumatic events at extremely young ages, it can be difficult for caregivers to discern whether behavioral changes have taken place. That is, it is often difficult for parents to recall whether a young child’s behavior changed from before to after witnessing a traumatic event, particularly if the parent also experienced trauma. In this case, the biological mother experienced interpersonal violence, which her daughter observed. During the parent interview, when asked about her child’s social behavior in the past, the mother reported that it was difficult for her to remember some events from that time period, including nuances of her daughter’s behaviors, due to her own trauma. This makes it difficult for mental health professionals to ascertain what patterns of behavior or personality may have existed prior to trauma (and therefore should not be considered as occurring “in reaction to” trauma), versus those that arose after the traumatic event.

### 3.7. Access and Barriers to Care

This family had financial stress, with an annual income of less than $15,000 per year. Due to its socio-economic status, the family had state-run insurance which made it difficult to find accessible mental health providers. This barrier to accessing care is particularly notable when trying to find professionals with a narrow and specific skill set (e.g., the ability to conduct a comprehensive assessment for ASD in light of a pre-existing diagnosis of ADHD and PTSD).

### 3.8. Follow-Up

Approximately 8 months after being evaluated, the patient’s mother was contacted to follow up on her daughter’s functioning. Unfortunately, her mother reported that symptoms had not improved, due to difficulty making notable progress in behavioral therapy. In addition, she reported concerns related to obtaining school-based services. In the USA, children who experience difficulty with education due to a disorder (either a physical disability or mental health diagnosis) are eligible for special education services. Children who do not require formal changes to the educational curriculum but require accommodations (e.g., a quiet testing environment and extra time for tests), receive a 504 plan. Children who require formal changes to the curriculum (e.g., different educational materials than other students), will have an Individualized Education Plan (IEP). Although the patient had a 504 plan (including accommodations for testing and extra time to complete assignments), she had been unable to receive an IEP for either Other Health Impairment or Emotional Disturbance [29], as she was performing at grade level in school. Her mother was concerned that emotional challenges and inappropriate behaviors were causing marked difficulty in school, and she was hopeful that the school would allow an IEP to be put in place in the next year or two. Overall, her mother was grateful to know that her daughter’s symptoms were not caused by ASD, as this allowed the family to seek targeted services related to trauma and attachment difficulties.

## 4. Discussion

This case study presented examples of two children who were seen in our autism clinic over the past year, in which the presenting question was the differential diagnosis of PTSD versus ASD. These cases highlight the complexity of accurately differentiating between symptoms of PTSD and ASD, particularly in young children who are unable to articulate their experiences.

### 4.1. Treatment Implications of the Cases

**Figure 1:**
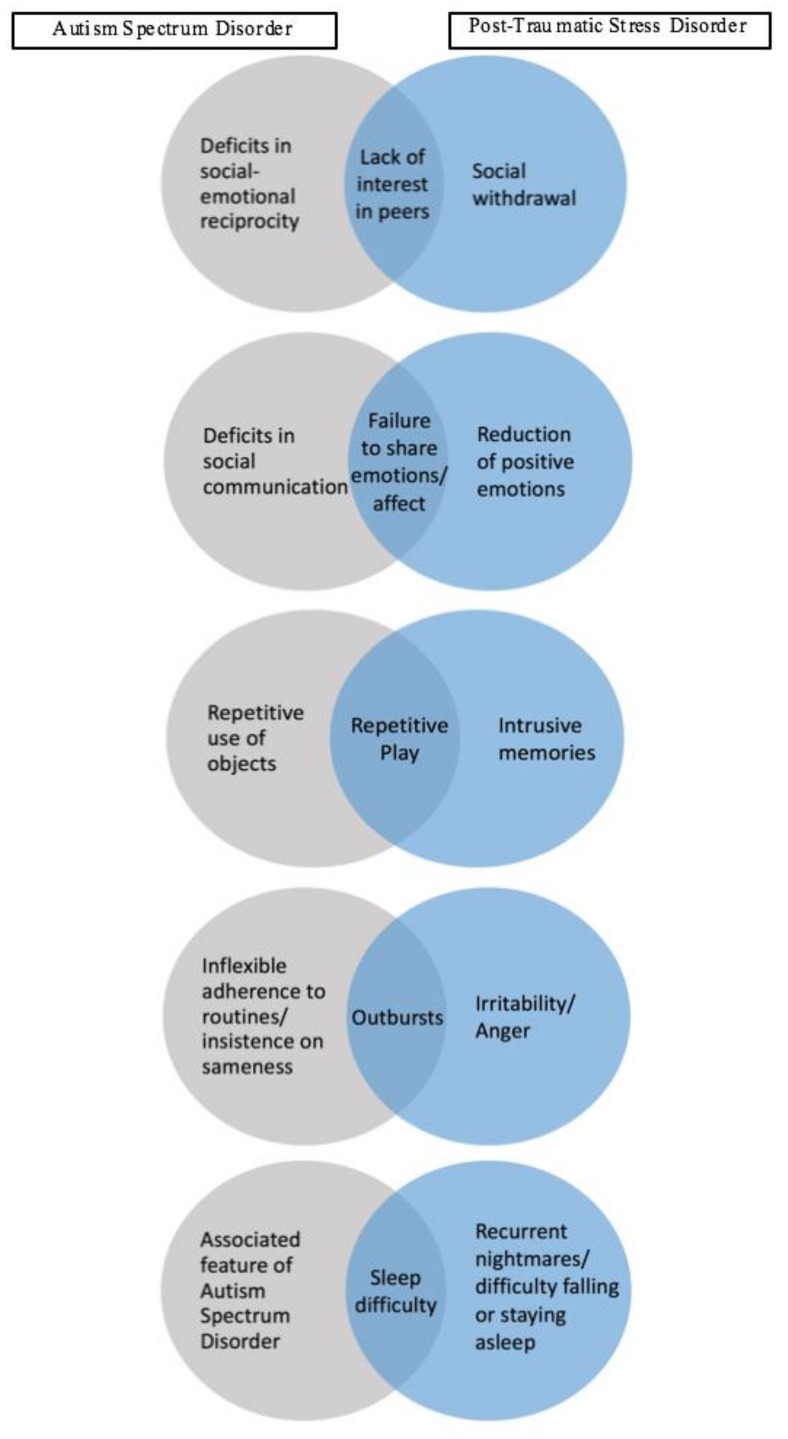
Diagram depicting underlying causes of observed behaviors in autism spectrum disorder and post-traumatic stress disorder.

The current cases emphasize how difficult it can be for professionals to attribute symptoms to one particular diagnosis in children, especially when symptom overlap is present between two disorders. In the two cases presented, social withdrawal, lack of social interest, and socially inappropriate behaviors were reported, which occur in PTSD, ASD, and other mood disorders. Figure 1 depicts some of the most prominent overlapping symptoms in PTSD and ASD. In Case 1, withdrawal was a primary concern, whereas Case 2 presented with lack of social boundaries and inappropriate social interactions. Although lack of social initiation is often associated with ASD [1], there are a variety of disorders which present with social withdrawal and lack of initiation. These include depression, social anxiety, and PTSD [30,31,32]). It is important for clinicians to consider multiple factors when determining if lack of social reciprocity is due to the social deficits observed in ASD or is due to PTSD.

First, it is helpful to establish a timeline of current behaviors via parent or caregiver interview. Children with ASD most likely do not display appropriate social initiation, and are often observed to be either disinterested in social interactions or have marked difficulty with age-appropriate social behavior, whereas children with PTSD are often noted to have changed at a certain age (e.g., after the traumatic event). This recommendation coincides with that of [18], who point out that symptoms such as restricted affect and social detachment are particularly difficult to differentiate between children with ASD and PTSD, and as such, clinicians should assess for changes in functioning rather than the presence or absence of symptoms in isolation. However, it is difficult to establish a timeline or assess change when children are exposed to trauma at a young age or when caregivers are unable to provide an historical report.

A second point of consideration is the quality of social interactions with the child (either via observation, interaction during the assessment, or by report). Children with ASD are likely to not only evidence reduced (or inappropriate/awkward) social initiation, but also have difficulties utilizing and integrating social communication skills. For example, children with ASD often display either reduced or poorly modulated eye contact, as well as less descriptive or functional gestures, and have difficulty integrating eye contact with gestures and speech. Children with PTSD, however, remain able to use social communication skills in tandem (e.g., eye contact with gestures and speech), but may display reductions in how often these skills are utilized, or with whom. It can therefore be helpful to ask caregivers, teachers, or siblings about how the child interacts when he or she is comfortable and familiar with someone, as this will differentiate between a child who rarely uses appropriate and integrated social communication skills and one who uses those skills less than before, but uses them appropriately when he or she feels safe and secure.

The presence of repetitive play was reported in Case 1, which is often associated with ASD, as one of the diagnostic criteria is the presence of restricted interests and repetitive behaviors [1]. However, in children with PTSD, repetitive play themes related to trauma are common [33], making it difficult to accurately classify repetitive play as a symptom of ASD versus PTSD. It is important for practitioners to note what types of play are being repeated, and whether the theme of play is repetitive (e.g., all play is related to a family in danger), or if the play actions themselves are repetitive (e.g., all play involves spinning wheels for prolonged periods of time, or use of objects in play is repetitive in nature). The former is more likely to be related to trauma, and the latter is akin to what is observed in children with ASD.

In terms of clinician interaction during assessments, we underscore the importance of utilizing standardized measures, in conjunction with parent interview and clinician judgement. Though the ADOS is considered the “gold standard” instrument for ASD diagnosis [34], it is important to keep in mind that ADOS specificity is lower in samples of children with mental health difficulties than in samples of children with developmental delays [35]. In the ADOS standardization sample, the specificity scores for children and adolescents with fluent language (e.g., Modules 3 and 4) is 0.93 and 0.94, respectively [36]. However, in a large sample of children and adolescents with mental health diagnoses other than ASD, specificity is 0.85 [35]. Overall, the ADOS is an extremely useful tool for diagnosis of ASD, but must be used in conjunction with other measures and clinician judgment.

### 4.2. Recommendations to Clinicians and Students

Taken together, the two cases reviewed underscore the importance of training clinicians and students to be aware of the overlap in symptoms of ASD and PTSD in children. Although the current case study provided examples of two children who came in for diagnostic clarification between PTSD and ASD, it is also important to note the difficulty of dual diagnosis. In the cases we reported, both children had PTSD rather than ASD, but there are many reports of children with ASD having difficulty obtaining a PTSD diagnosis due to clinical overshadowing and the assumption that all of their symptoms are related to ASD. The issue of accurate dual diagnosis is of particular importance due to the increased risk of maltreatment in children with ASD, compared to their typically-developing peers. As noted by Brenner and colleagues [4], there is substantial evidence that children with developmental disabilities, including ASD, are between 1.6 and 2.2 times more likely to experience neglect, physical abuse, or sexual abuse. Studies of children with ASD, in particular, suggest that children with ASD involved in Child Protective Services (CPS) are 3–4 times more likely to be maltreated than other children involved with CPS. These findings underscore the importance of providing training to clinicians and students in medical, psychology/psychiatry, education, and other related fields with regards to the overlap of symptoms between ASD and PTSD, how to provide an accurate differential diagnosis, and when a dual diagnosis is warranted.

Finally, despite the overlap in symptoms of PTSD and ASD, the recommended treatments are markedly different, which further emphasizes the importance of an accurate diagnosis. For ASD, behavioral interventions focus on increasing social communication skills and decreasing problematic behaviors. Applied Behavioral Analysis (ABA) and Early Start Denver Model (ESDM) are two of the most widely known and studied interventions, but there are a variety of evidence-based programs for children with ASD (e.g., [37,38]). Treatments for PTSD in children, on the other hand, is often cognitive behavioral therapy (CBT), which focuses on psychoeducation, relaxation and desensitization strategies, and cognitive reframing, or CBT combined with pharmacological intervention [39]. It is difficult to accurately diagnose young children with psychological disorders, as they are often unable to describe and explain their own thoughts, feelings, and behaviors. However, accurate and early diagnosis allows for targeted treatment strategies, which can alleviate symptoms and help children live more fulfilling lives.
